# Rewiring tumor-associated macrophages in hepatocellular carcinoma

**DOI:** 10.3389/fimmu.2026.1775603

**Published:** 2026-01-27

**Authors:** Xue Han, Rui Jin

**Affiliations:** 1 Department of Gastroenterology, The Affiliated Huai’an Hospital of Xuzhou Medical University, Huai’an, China; 2 Department of Gastroenterology, Huaian 82 Hospital, Huai’an, China

**Keywords:** hepatocellular carcinoma, immune evasion, immunotherapy, polarization, tumor-associated macrophages

## Abstract

Hepatocellular carcinoma (HCC) remains a leading cause of cancer-related mortality worldwide, with limited responsiveness to immune checkpoint inhibitors (ICIs). Accumulating evidence indicates that tumor-associated macrophages (TAMs) are central regulators of the immunosuppressive tumor microenvironment (TME) and major contributors to immune escape and therapeutic resistance in HCC. TAMs arise from both circulating monocytes and tissue-resident macrophages and exhibit remarkable plasticity, adopting diverse polarization states in response to microenvironmental cues. Beyond the classical M1/M2 paradigm, single-cell and spatial technologies have revealed a continuum of TAM phenotypes with distinct transcriptional, metabolic, and functional properties. These heterogeneous TAM subsets orchestrate angiogenesis, fibrosis, immune suppression, and resistance to immunotherapy. Consequently, TAMs have emerged as attractive therapeutic targets. Strategies aimed at limiting monocyte recruitment, reprogramming M2-like TAMs toward antitumoral phenotypes, exploiting TAMs as drug delivery vehicles, and combining TAM-targeted interventions with ICIs, radiotherapy, anti-angiogenic agents, or nanobiotechnology have shown promising preclinical and early clinical efficacy. This review summarizes current advances in understanding TAM origin, polarization heterogeneity, and functional roles in HCC, and highlights emerging TAM-centered therapeutic strategies that may improve immunotherapy outcomes and enable more precise, durable treatment responses.

## 1 Introduction

Hepatocellular carcinoma (HCC), the most prevalent form of primary liver cancer globally (1–3), has seen a paradigm shift in treatment with PD-L1–based combination immunotherapy now serving as the important option for patients with unresectable disease (4, 5). Despite initial efficacy, a substantial subset of patients rapidly develop resistance to immune checkpoint inhibitors (ICIs), undermining long-term therapeutic benefit. Emerging evidence identifies tumor-associated macrophages (TAMs) as central mediators of ICI resistance in HCC (6). The tumor microenvironment (TME) is a dynamic and multifaceted ecosystem comprising immune cells, stromal elements, vasculature, and extracellular matrix which plays an essential role in regulating tumor initiation, invasion, angiogenesis, metastasis, and immune evasion (7–10).

TAMs constitute a dominant immune subset, originating from circulating monocytes or tissue-resident macrophages recruited into the tumor site (11, 12). Functionally, TAMs act as a critical immunosuppressive barrier, exhibiting phenotypic plasticity and contributing to both tumor progression and immune tolerance (13). TAMs have been implicated in promoting angiogenesis, inflammation, epithelial–mesenchymal transition (EMT), and immune escape (14, 15). Therefore, targeting TAMs has emerged as a promising strategy in antitumor immunotherapy (16). In HCC, TAMs not only mediate immune escape and resistance to immunotherapy but also participate in tumor initiation and progression via diverse mechanisms (17, 18). Consequently, exploring the functional and molecular landscape of TAMs in HCC may offer new therapeutic insights and provide a theoretical basis for the development of TAM-targeted antitumor therapies.

## 2 Polarization and functional roles of TAMs

### 2.1 Roles of M1 TAMs in HCC

M1 macrophages are typically activated by pathogen- or damage-associated molecular patterns (PAMPs/DAMPs), including bacterial lipids such as lipopolysaccharide (LPS), oxidized low-density lipoprotein (oxLDL), viral nucleic acids, IFN-γ, TNF-α, and granulocyte-macrophage colony-stimulating factor (GM-CSF) (19, 20). These stimuli engage Toll-like receptors (TLRs), particularly TLR4, initiating a proinflammatory transcriptional program (21). Upon stimulation, they secrete large amounts of pro-inflammatory cytokines, including interleukins and TNF-α, thereby exerting potent inflammatory responses. In addition, M1 macrophages induce inducible nitric oxide synthase (iNOS) expression, leading to nitric oxide (NO) production and the release of reactive oxygen species (ROS), which are cytotoxic to tumor cells (22, 23). In hepatocellular carcinoma, M1-type TAMs can inhibit tumor cell proliferation through multiple mechanisms. For example, activation of SIRT1 suppresses NF-κB signaling, thereby reducing inflammatory responses and hepatocarcinogenesis (24, 25). Furthermore, upregulation of retinoic acid-inducible gene I (RIG-I) expression can activate the RIG-I/MAVS/TRAF2/NF-κB signaling axis, enhancing immune surveillance and promoting antitumor immunity in murine HCC models (26, 27). Additionally, M1-polarized TAMs can inhibit HCC cell proliferation by producing IL-12, which suppresses STAT3 and c-Myc signaling pathways, ultimately impairing cell cycle progression and tumor growth (28, 29).

### 2.2 Mechanisms of M2 TAMs in HCC

**Figure 1:**
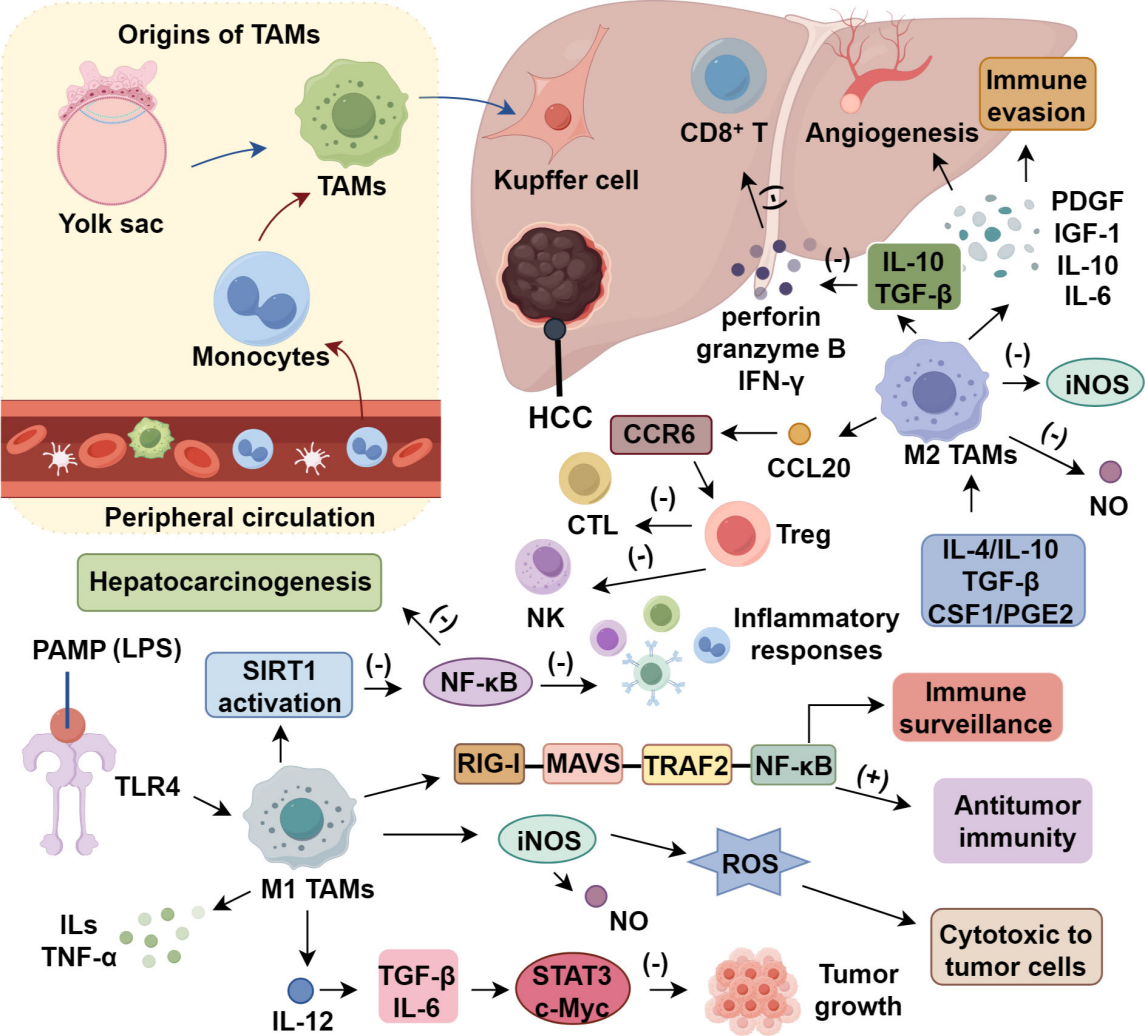
Tumor-associated macrophages (TAMs) in hepatocellular carcinoma.

M2 macrophages, typically activated by Th2 cytokines (IL-4, IL-10, IL-13), TGF-β, CSF1, and tumor-derived factors such as PGE2, exhibit immunosuppressive and pro-tumoral functions distinct from M1 macrophages (30, 31). Upon polarization, M2-like TAMs downregulate iNOS and NO production while secreting growth factors (PDGF, IGF-1) and cytokines (IL-10, IL-6, TNF-α, CCL2) that promote angiogenesis, tissue remodeling, and tumor immune evasion (17, 32). In HCC, TAMs have been shown to induce epithelial–mesenchymal transition (EMT) through secretion of TGF-β and IL-6, which activate the STAT3 and TGF-β/Smad pathways in hepatoma cells, facilitating invasion and metastasis (33, 34). They inhibit Th1-mediated cytotoxicity and shift the immune balance toward a Th2-dominant environment, facilitating tumor growth and metastasis (33). Notably, IL-10 and TGF-β secreted by M2 TAMs suppress CD8^+^ T cell cytotoxicity by inhibiting the expression of perforin, granzyme B, and IFN-γ, thereby impairing effector T cell function and limiting antitumor immunity (35, 36). IL-10 also promotes the differentiation of naïve CD4^+^ T cells into regulatory T cells (Tregs), reinforcing the immunosuppressive microenvironment. Meanwhile, TGF-β induces Smad-dependent signaling in CD8^+^ T cells, leading to reduced proliferation and diminished cytotoxic granule release (37, 38). TAM-derived CCL20 interacts with CCR6 receptors on Tregs, promoting their chemotactic recruitment and local expansion within the tumor microenvironment (39, 40). This accumulation of Tregs further inhibits the activity of cytotoxic T lymphocytes and natural killer (NK) cells, thereby enhancing immune evasion in HCC (41, 42). In advanced fibrotic HCC, CCR2^+^ TAMs play a pivotal role in maintaining tumor vasculature, and their depletion disrupts vessel integrity (43). Th2-polarized CD4^+^ T cells secrete IL-23, which binds to IL-23 receptors (IL-23R) expressed on macrophages, thereby promoting M2 polarization (44). IL-23/IL-23R signaling activates STAT3 phosphorylation in TAMs, driving transcriptional programs associated with alternative activation and immunosuppressive cytokine production (IL-10, TGF-β) (31, 45). Additionally, IL-23–induced STAT3 activation may facilitate the recruitment or maintenance of regulatory T cells (Tregs) within the HCC tumor microenvironment by upregulating Treg-supportive chemokines (CCL22) and sustaining immune tolerance (45, 46). This axis thereby links Th2 immunity with both macrophage-mediated immunosuppression and Treg-driven immune evasion. IL-23 produced by Th2-polarized CD4^+^ T cells enhance IL-23 receptor expression in TAMs, further promoting M2 polarization (44). M2 macrophages can be subdivided into M2a, M2b, and M2c, all contributing to an immunosuppressive tumor microenvironment and resistance to therapy (17, 47) (**Figure 1**).

### 2.3 Heterogeneity of TAM polarization

Recent studies have revealed that macrophage polarization is far more complex than the classical M1/M2 dichotomy (48). TAMs exhibit a spectrum of activation states with overlapping features, and may simultaneously express both M1 and M2 markers, reflecting their dual roles in pro-inflammatory and immunosuppressive processes. With the advancement of technologies such as single-cell RNA sequencing and mass cytometry, multiple TAM subpopulations with distinct transcriptomic and functional profiles have been identified (49). In HCC, single-cell transcriptomic analyses have identified novel TAM subsets with distinct immunoregulatory roles (50). Notably, SPP1^+^ macrophages are enriched in hypoxic and fibrotic regions of tumors and exhibit strong pro-angiogenic and matrix-remodeling signatures (51, 52). MARCO^+^ TAMs, characterized by high lipid metabolism and scavenger receptor expression, have been associated with T cell exclusion and immunotherapy resistance. TREM2^+^ macrophages demonstrate potent immunosuppressive functions and correlate with poor clinical outcomes in HCC (53–55). M1-like TAMs tend to express pro-inflammatory genes such as FCGR3A, IL-12, and TNF, whereas M2-like TAMs upregulate tumor-supportive genes including TGF-β, VEGFA, PDGF-B, IL-6, IL-1β, and chemokines such as CCL2 (33, 56). Highly proliferative M2-like TAMs express markers such as CD206, MKi67, and CD163, and are enriched in fibrotic and angiogenesis (57). In contrast, inflammatory TAMs are more abundant in tumor-surrounding tissues. Additionally, myeloid-derived suppressor cells (MDSCs) share overlapping features with M2-like TAMs (23, 58). These cells express immunosuppressive genes such as THBS1, FCN1, VCAN, and CD33, and are associated with immune evasion and tumor progression (59). M2-TAMs and MDSCs dominate within the tumor core, while pro-inflammatory TAMs are more frequently observed at the tumor margin.

## 3 TAMs in immunotherapy of HCC

TAMs are central regulators of the tumor microenvironment (TME), modulating immune responses and facilitating tumor progression. Consequently, TAMs have emerged as promising targets for cancer immunotherapy (60). Current therapeutic strategies include: inhibition of monocyte recruitment, reprogramming of TAMs toward pro-inflammatory M1-like states, employing TAMs as drug delivery vectors, and combining TAM-targeting approaches with immune checkpoint blockade (61, 62). Monocyte recruitment into tumors is predominantly mediated through chemokine–receptor axes such as CCR2/CCL2, CX3CR1/CX3CL1, CCL3/4/5, and VEGF-A/VEGFR1 (63). Pharmacological disruption of these pathways—particularly targeting CCR2, M-CSF, or CSF1R—effectively reduces monocyte infiltration, limits M2-like TAM polarization, and alleviates immunosuppressive TME conditions (29, 64, 65). Notably, CCL2–CCR2 blockade decreases TAM density and M2-associated cytokines (IL-10, TGF-β), thereby fostering an immune-permissive milieu (66). Combined inhibition of CCR2 or CSF1R with immune checkpoint blockade, such as anti–PD-1 or anti–CTLA-4 therapies, has demonstrated synergistic antitumor effects. For instance, PLX3397 (pexidartinib) enhances checkpoint efficacy in preclinical models (67, 68), while RS102895 (a CCR2 antagonist) augments T cell infiltration, reduces PD-L1^+^ TAMs, and restores MHC-II–mediated antigen presentation in hepatocellular carcinoma and lung cancer models (69). Furthermore, targeting specific TAM subsets based on surface markers (CX3CR1) offers additional therapeutic avenues. CX3CL1 secreted by hepatic stellate cells recruits CX3CR1^+^ TAMs that suppress CD8^+^ T cell cytotoxicity and promote immune evasion (70). Inhibiting this axis not only curtails TAM accumulation but also synergizes with PD-1 blockade to restore cytotoxic T lymphocyte (CTL) function and suppress tumor growth (71).

### 3.1 Reprogramming of TAMs

TAMs possess high plasticity and can be reprogrammed between M1- and M2-like phenotypes. M1-polarized TAMs primarily exert anti-tumor functions during early stages of hepatocarcinogenesis, while M2-like TAMs, induced by chronic inflammation and hypoxia, promote tumor progression and metastasis (29, 72). Thus, re-educating TAMs toward an M1 phenotype has emerged as a promising therapeutic avenue. One approach involves blocking Toll-like receptor 9 (TLR9) signaling, which reverses mtDNA-induced M2 polarization, thereby enhancing sorafenib efficacy in HCC (73). Galectin-1 (Gal-1), secreted by hepatic stellate cells, promotes TAM differentiation into Treg-inducing M2 cells. Gal-1 inhibition reduces CCL20 production, suppresses monocyte recruitment, and thereby enhances tumor immunogenicity and response to PD-1 checkpoint blockade therapy (74). In a murine orthotopic liver cancer model, dual blockade of GPC3 and CD47 significantly increased M1/M2 TAM ratios and reduced overall TAM density, indicating that GPC3/CD47-targeted antibodies can shift TAMs toward a pro-inflammatory phenotype and enhance anti-tumor immunity (75). The CSF1–CSF1R signaling axis is closely linked to TAM accumulation and polarization. Inhibitors such as PLX3397 (pexidartinib) can suppress TAM recruitment and repolarize them toward M1-like phenotypes, effectively inhibiting liver tumor progression (76). Other agents such as STAT3 inhibitors, PI3K blockers, JAK/STAT pathway modulators, miRNA mimics or inhibitors (miR-155, miR-21-5p, lncRNA-NEAT1) have also shown efficacy in reprogramming M2 TAMs into an inflammatory, tumoricidal phenotype (77–83). STAT3 and NF-κB signaling pathways are recognized as classical regulators of TAM plasticity and immune suppression in HCC (84). Persistent activation of STAT3 in TAMs promotes M2 polarization by enhancing IL-10 and VEGF secretion, thereby suppressing antigen presentation and dampening T cell responses (85). Conversely, pharmacologic inhibition of STAT3 can shift TAMs toward M1-like phenotypes and restore antitumor immunity (86, 87). Similarly, the NF-κB pathway, while generally associated with inflammatory responses, can paradoxically promote tumor progression when aberrantly activated in TAMs (88). In HCC models, NF-κB–driven upregulation of IL-6 and S100 family proteins contributes to chronic inflammation and tumor-supportive functions (89, 90). Therefore, modulating these canonical pathways remains a viable and complementary approach to TAM-directed therapies.

### 3.2 Nanomedicine-based TAM therapies

Recent progress in macrophage-targeted nanomedicine has shifted focus toward reprogramming TAMs through encapsulated agents that modulate intracellular signaling (91). Nanoparticles increasingly function not merely as drug carriers, but as immunoregulatory tools capable of reshaping the tumor microenvironment. They can promote ROS production, thereby activating redox-sensitive transcription factors such as NF-κB. Additionally, engagement of Toll-like receptors (TLR7, TLR8, and TLR9) triggers proinflammatory cascades, while mitochondrial stress responses rewire macrophage metabolism toward a glycolytic, proinflammatory state (92, 93). These mechanisms highlight a growing paradigm in which nanomaterials serve as direct modulators of innate immune function within tumors. Despite inherent limitations in tumor penetration, M1-like TAMs exhibit intrinsic tumor-homing capacity and prolonged retention in tumor tissues, rendering them attractive candidates for therapeutic delivery (94, 95). Nanoparticles engineered to mimic macrophage membranes preferentially accumulate in tumor sites and enhance immunotherapeutic efficacy through targeted payload release. For example, MnO_2_-loaded sorafenib nanoparticles utilize TAMs as delivery vehicles. Within the tumor microenvironment, MnO_2_ reacts with endogenous hydrogen peroxide (H_2_O_2_) to release Mn²^+^, enhancing sorafenib delivery, improving CD8^+^ T cell infiltration, increasing PD-1 blockade responsiveness, and suppressing tumor growth in HCC models (96). CDNP-R848, a TLR7 agonist-loaded β-cyclodextrin nanoparticle formulation, which reprograms M2-TAMs into inflammatory M1 phenotypes and enhances innate immune activation (97, 98). Besides, HA-PEI nanocarriers loaded with microRNA-125b have been shown to reprogram M2-like TAMs into M1-like states, thereby reversing immunosuppression and amplifying antitumor responses (99–101). These strategies demonstrate the therapeutic synergy of nanotechnology and TAM reprogramming, offering precise control over macrophage plasticity and immune modulation within the tumor microenvironment.

### 3.3 Combination therapies

TAMs possess substantial heterogeneity and plasticity, dynamically adapting to fluctuating signals within the tumor microenvironment (102). This complexity presents a challenge for therapeutic interventions that aim to modulate TAMs without disrupting essential immune or stromal homeostasis. Recent efforts have focused on reprogramming or depleting immunosuppressive TAM subsets while combining these strategies with other modalities, such as radiotherapy (RT), chemotherapy, or immune checkpoint inhibitors, to enhance treatment efficacy and mitigate TME-driven therapeutic resistance (103–105). In HCC, TAM density has been proposed as a predictive biomarker for radiotherapy responsiveness (106). RT can induce IL-4 and CSF1 signaling, which facilitates M2 polarization and monocyte recruitment, potentially diminishing therapeutic efficacy. However, combination strategies, such as RT plus CSF1R inhibitors, have shown promise in reversing this resistance (107). For instance, RT combined with IL-12 therapy enhances MHC-II and co-stimulatory molecule (CD40, CD86) expression on dendritic cells, limits MDSC accumulation, and reduces ROS production, collectively improving antitumor immunity. In HCC, RT combined with IL-12 has been shown to stimulate TAM-mediated immune reprogramming and promote T cell activation, ultimately reversing immune evasion and reducing tumor burden (108). Nonetheless, the impact of TAM phenotypic plasticity and the temporal coordination between TAM-targeting and RT remain incompletely understood, warranting further investigation. TAMs contribute to both extracellular matrix (ECM) remodeling and the preservation of cancer stemness. By promoting matrix degradation and sustaining an immunosuppressive microenvironment, TAMs enhance and progression (109–112). They also promote resistance by activating STAT3, IL-6, and TGF-β signaling pathways, supporting tumor cell survival and stem-like traits (113–115) (**Table 1**).

**Table 1:** Immunotherapy strategies targeting TAMs in HCC.

Strategy	Mechanism	Representative agents	Function
Inhibition of Monocyte Recruitment	Blocks chemokine signaling to prevent TAM infiltration into tumor tissue	CCR2 inhibitors (RS102895), CSF1/CSF1R blockade (PLX3397)	Decreases M2 TAM infiltration, restores antitumor immune responses, enhances immune checkpoint blockade efficacy
Reprogramming M2 to M1 TAMs	Induces functional shift from pro-tumoral to inflammatory phenotype	TLR9 inhibitors, Galectin-1 inhibitors, STAT3/PI3K/JAK pathway blockers, miR-155, miR-23b-3p, lncRNA-0243	Boosts CD8^+^ T cell activity, reduces immunosuppressive environment
TAM-Targeted Drug Delivery	Selective delivery of therapeutics using TAM-specific markers or nanocarriers	MnO_2_-sorafenib nanoparticles, CDNP-R848 (TLR7 agonist), HA-PEI-microRNA nanocarriers	Increases intratumoral drug accumulation, remodels tumor microenvironment
Combination with Immune Checkpoint Inhibitors	Enhances response to anti-PD-1/PD-L1 or CTLA-4 therapy	Anti-PD-1 combined with CCR2 or CSF1R inhibitors, GPC3/CD47 bispecific antibodies	Induces synergistic tumor regression, promotes antigen presentation
Combination with Radiotherapy	Counteracts radiation-induced TAM immunosuppression	Radiotherapy with IL-12, Radiotherapy with CSF1R inhibitors	Enhances MHC-II and costimulatory molecule expression on TAMs, reduces MDSCs
Anti-Angiogenic Therapy	Modulates TAM function by targeting VEGF/FGFR pathways	TKIs (Lenvatinib, Sorafenib, Apatinib), VEGFR2 inhibitors	Facilitates M1 polarization, suppresses metastasis, augments immunotherapy
CAR-Macrophage Therapy	Employs engineered macrophages with tumor-targeting specificity	CAR-M (GPC3-specific chimeric antigen receptor macrophages)	Enhances phagocytosis, activates adaptive immunity, promotes immune activation

### 3.4 Anti-angiogenic therapy and CAR-M

Anti-angiogenic agents can inhibit TAM polarization toward an M2-like phenotype or even reprogram M2 TAMs into M1-like macrophages (116, 117). Tyrosine kinase inhibitors (TKIs)—such as sorafenib, regorafenib, and lenvatinib—not only exert direct anti-angiogenic effects but also reshape the immune microenvironment by modulating TAM function and promoting systemic antitumor immunity (118, 119). Lenvatinib, for example, inhibits the p38 MAPK/NF-κB signaling pathway, leading to downregulation of TNF-α and IL-6 in HCC mouse models (120). This reduces tumor metastasis and enhances M1-like TAM polarization. When combined with PD-1 blockade, lenvatinib increases CD8^+^ T cell infiltration and promotes a proinflammatory immune milieu (121, 122). Furthermore, lenvatinib downregulates FGFR signaling and enhances antigen presentation and PD-1-mediated T cell activation, reinforcing the efficacy of checkpoint inhibitors (123). In parallel, apatinib has been shown to reduce TAM-induced PD-L1 expression in tumor cells and to suppress MDSC accumulation within the TME, even at low doses (124). These findings highlight the therapeutic synergy between anti-angiogenic agents and immunotherapies, offering new opportunities for combinatorial cancer treatment. VEGFR blockade by tyrosine kinase inhibitors (TKIs) such as lenvatinib or apatinib exerts multifaceted immunomodulatory effects by altering TAM polarization through key signaling pathways (121, 125). Suppression of VEGF/VEGFR signaling reduces hypoxia-inducible factor 1-alpha (HIF-1α), a transcriptional regulator critical for maintaining the M2-like, pro-angiogenic TAM phenotype under hypoxic conditions (126–128). This downregulation diminishes expression of genes including ARG1 and VEGFA, thereby impairing M2 polarization and facilitating M1-like reprogramming (129, 130). Concurrently, VEGFR inhibition attenuates STAT3 activation, a pivotal inducer of M2-associated cytokines such as IL-10 and TGF-β. Reduced STAT3 signaling enhances proinflammatory cytokine production (IL-12, TNF-α) and antigen-presenting capacity (131, 132). In addition to pharmacological strategies, cell-based immunotherapy is emerging as a novel direction. Pre-clinical trials have explored the application of chimeric antigen receptor macrophages (CAR-M) in solid tumors, including HCC (133, 134). CAR-Ms are genetically engineered to express tumor-specific antigen receptors, enabling them to phagocytose tumor cells while secreting proinflammatory cytokines that reshape the TME (135). Moreover, CAR-Ms have demonstrated the ability to reverse M2-like immunosuppression and stimulate systemic antitumor responses, positioning them as a next-generation cellular platform for immunotherapy (136, 137).

## 4 Conclusion

Tumor-associated macrophages (TAMs) are integral architects of the immunosuppressive tumor microenvironment in hepatocellular carcinoma (HCC), mediating immune escape, therapy resistance, angiogenesis, and metastasis. Recent advances in single-cell transcriptomics and spatial profiling have uncovered the remarkable heterogeneity and plasticity of TAMs beyond the classical M1/M2 framework, revealing novel subsets such as SPP1^+^, MARCO^+^, and TREM2^+^ macrophages with distinct functional roles. These insights have redefined TAMs not only as biomarkers of disease progression but also as versatile therapeutic targets capable of being reprogrammed to enhance antitumor immunity.

Targeting TAMs offers multiple complementary opportunities to overcome immune resistance in HCC. Strategies that inhibit monocyte recruitment, suppress M2 polarization, or actively reprogram TAMs toward M1-like phenotypes have demonstrated the capacity to restore cytotoxic T cell function and enhance responsiveness to ICIs. Moreover, the integration of TAM modulation with radiotherapy, anti-angiogenic agents, tyrosine kinase inhibitors, and nanotechnology-based delivery systems has uncovered synergistic effects that reshape the immunosuppressive TME. Emerging cellular approaches, such as chimeric antigen receptor macrophages, further expand the therapeutic landscape by harnessing macrophage phagocytosis and antigen presentation. Despite these advances, key challenges remain, including defining optimal TAM subsets to target, timing of intervention, and minimizing off-target immune perturbations. Future translational studies integrating multi-omics profiling, spatial biology, and rational combination strategies will be essential to fully exploit TAM-directed therapies and advance precision immunotherapy for HCC.
